# Environmental Trigger(s) of Type 1 Diabetes: Why So Difficult to Identify?

**DOI:** 10.1155/2015/321656

**Published:** 2015-03-25

**Authors:** Kjersti S. Rønningen

**Affiliations:** Department of Pediatric Research, Division for Women and Children, Oslo University Hospital, Rikshospitalet, 0027 Oslo, Norway

## Abstract

Type 1 diabetes (T1D) is one of the most common chronic diseases with childhood onset, and the disease has increased two- to fivefold over the past half century by as yet unknown means. T1D occurs when the body's immune system turns against itself so that, in a very specific and targeted way, it destroys the pancreatic *β*-cells. T1D results from poorly defined interactions between susceptibility genes and environmental determinants. In contrast to the rapid progress in finding T1D genes, identification and confirmation of environmental determinants remain a formidable challenge. This review article will focus on factors which have to be evaluated and decision to take before starting a new prospective cohort study. Considering all the large ongoing prospective studies, new and more conclusive data than that obtained so far should instead come from international collaboration on the ongoing cohort studies.

## 1. Introduction

Type 1 diabetes (T1D) is one of the most common chronic diseases with childhood onset, and the disease has increased two- to fivefold over the past half century by as yet unknown means [1, 2]. It was interpreted that if present trend continues, the prevalence of children with the disease in Europe will increase with 50% within year 2020 and with 70% for those less than 5 years of age [3]. Most recently Europe was compared for the periods 1989–1998 and 1999–2008, and it was shown that the increase in T1D is not necessary uniform, showing periods of less rapid and more rapid increase in incidence for different countries [4]. This pattern of change suggests that important risk exposure differs over time in different European countries.

T1D occurs when the body's immune system turns against itself so that, in a very specific and targeted way, it destroys the pancreatic islet *β*-cells, the only cells in the body that produce the vital hormone insulin [5, 6]. This autoimmune destruction is irreversible and the disease is incurable. If pancreas or islets are transplanted they too are destroyed, unless heavy immunosuppression is applied [7].

T1D results from poorly defined interactions between susceptibility genes and environmental determinants. T1D susceptibility is primary defined by genetic factors within the human leukocyte antigen (HLA) on chromosome 6. The main disease factors are the HLA-DQ molecule encoded by DQA1 and DQB1 genes and the HLA-DR molecule defined by DRB1 alleles [8–12]. In addition, recent genome-wide association studies have identified more than 40 other intervals that may harbour T1D susceptibility genes [13].

In contrast to the rapid progress in finding T1D genes, identification and confirmation of environmental determinants remain a formidable challenge [14]. The reason underlying the lack of progress is multifaceted. First, different categories and large numbers of environmental determinants could contribute to the triggering or protection of T1D [15–38]. Although many candidates have been suggested in the past, few have been definitively proven beyond reasonable doubt. Second, exposures may occur any time before the onset of disease, from in utero to T1D onset [39–43]. Third, environmental determinants may differ in different populations, partly depending on the genetic architecture. Fourth, the individual risk of developing T1D in the general population is not very high and quite variable in different populations. Therefore, large study populations with elevated T1D risk must be identified.

Islet autoantibodies precede the development of T1D and can appear throughout childhood [44]. In prospective studies of offspring of parents with T1D, a peak incidence of islet autoantibodies appearance at around 1 year of age has been observed, followed by a decline through 2–5 years and a subsequent rise in incidence towards puberty [45]. Children with increased HLA-associated risk for T1D followed up from the general population get positive for autoantibodies at all ages. Early seroconversion occurs in children who progress fast to T1D [46]. It has been observed that, in children who progress to T1D during a follow-up of 13 years, 64% became autoantibody positive before the age of 2 years and 82% before the age of 3 years [46]. Early antibody positivity frequently starts with insulin autoantibodies (IAA) followed by glutamic acid decarboxylase antibodies (GADA). Usually insulinoma-associated protein 2 (IA-2) and zinc transporter 8 (ZnT8) antibodies develop closer to onset of T1D [45].

Identification of environmental determinants requires frequent follow-up for autoantibody testing and a large number of individuals from early in life until disease onset for studying a variety of exposures using epidemiological and laboratory methodologies. To accomplish such ambitious goals, long-term prospective studies on cohorts of children at increased risk of developing the disease are necessary. To design the study as good as possible is of most importance to achieve the identification of important environmental factor before recruitment starts. The MIDIA study will be used for the discussion of my own and my group experiences.

## 2. The MIDIA Study

### 2.1. Which HLA Class II Genes to Type for?

Based on the hypothesis that it should be most easy to identify the environmental trigger(s) among children with the highest genetic risk and that this limited number of children for follow-up would make it possible to recruit all over Norway with a centralized laboratory and working staff in Oslo, the decision for how to recruit and run the MIDIA (Norwegian abbreviation for Environmental trigger(s) of type 1 diabetes) study was taken. It is approximately 60,000 births in Norway per year, and only 2.2% of all babies carry the highest genetic risk for T1D (e.g., the HLA-DR3-DQ2/DR4-8 (DRB1^*^ 03-DQA1^*^ 05-DQB1^*^ 02/DRB1^*^ 04:01-DQA1^*^ 03-DQBI^*^ 03:02/03:04) genotype).

### 2.2. Follow-Up in MIDIA

The aim of MIDIA was to genotype 100,000 babies to achieve 2000 children for follow-up for 15 years. Such babies have 7% risk for getting T1D before 15 years of age and a life long risk at 20%, compared to 0.4% risk before 15 years of age and lifelong at 1% for newborns of the general population. A special MIDIA computer program was made before the start of recruitment and covered recruitment (reports were made and sent out twice per year for the status of the 638 communities and 19 counties in Norway); and a certain incoming sample had a well-defined place in the laboratory tracking system. In addition the MIDIA program could analyze real-time PCR results and conclude for a certain HLA gene. One list showed letters to parents not having a child at the highest risk for T1D, and another list showed other parents that should be called because the genotyping had shown that they had a child with the highest genetic risk. Such children did also come on lists for getting a second call (how the families were dealing with the high-risk information, and if they had more questions). The program was and is still responsible for follow-up of high-risk children. The time point for sending out all follow-up packages, the content for a certain package, and the time for sending it out are told. The program also follows data for autoantistoff positivity, and with positivity for one autoantibody it is told by the program to send out a package 6 months after the incoming result and for two or three autoantibodies to send out a new package for blood drawing after 3 months; see Table 1.

With the aim of performing HLA class II typing to identify the highest risk genotype—DQB1^*^ 04:01-DQB1^*^ 03:02/04/DR3-DQA1^*^ 05-DQB1^*^ 02—as easy and robust as possible, it was decided to use a four-step strategy [47]. Albumin with monomorphic primers was used in the polymerase chain reaction (PCR) to show if a sample contained enough DNA to be genotyped successfully. Sequence-specific reactions for all samples were performed for DQA1^*^ 03 and -^*^ 05. When both DQA1^*^ 05 and DQA1^*^ 03 were identified for a certain sample, the next step was DQB1 sequence-specific typing. The identification of DQB1^*^ 03:02/04 and DQB1^*^ 02 brought the sample to the third step in the typing protocol. DRB1^*^ 4 subtyping was performed for all samples positive for the DQA1^*^ 03-DQB1^*^ 03:02/04 haplotype. All different DR4 subtypes were positively identified, but only the DRB1^*^ 04:01 gene is in Norwegian conferring the highest risk [11, 12]. All samples found positive for the highest risk genotype (DRB1^*^ 04:01-DQA1^*^ 03-DQB1^*^ 03:02/4/DRB1^*^ 03-DQA1^*^ 05-DQB1^*^ 02) went to a confirmatory step (e.g., step 4) through all 3 steps in the typing protocol once more together with the most recent incoming DNA samples for typing. This strategy of confirmation detected only two errors during the genotyping in MIDIA (2001–2007). The parents of the babies where this happened had already received a nonrisk letter, since such letters were sent out daily for samples not fulfilling the demands to go further in the MIDIA genotyping. Luckily both two parent pairs understood the explanation given to them, and they joined the follow-up for high-risk children in MIDIA.

### 2.3. Inclusion Criteria

Mothers of preterm babies as well as those who had got a child with malformations did not receive invitation to MIDIA participation. For being eligible for participation at least one of the parents needed to be of Caucasian origin. Asian and African people do not carry the DRB1^*^ 0401-DQB1^*^ 03-DQB1^*^ 03:02/4 haplotype [9, 48]. Responsible for recruitment, and in most cases for taken a buccal sample of the baby, was a public health care nurse that in advance had got updated knowledge on T1D and learned how to inform about genetic risk for T1D by the Principal Investigator (PI) of the study and a study coordinator (a public health care nurse working closely together with the PI of MIDIA at the Norwegian Institute of Public Health).

### 2.4. All over Norway

All contact with the participating families had to take place by phone calls since there was never enough funding to bring participating families to the Norwegian Institute of Public Health. The distance from Kirkenes in the North-Eastern Norway is the same as the distance from Oslo to Rome, Italy. From the beginning of the study, there were less than 5% of parents informed about genetic high-risk for their baby that did not want to take part in the follow-up. Stool samples were asked for from the baby for 3–35 months of age and blood samples at 3, 6, and 9 and 12 months, and thereafter annually. Questionnaires were asked for at the same time point as blood samples. Until 2006 we got 94% of all stool samples, 83% of all questionnaires, and 86% of all blood samples that we asked for [49].

### 2.5. Stopping of an Ongoing T1D Study Based on the Norwegian Biotechnology Law

The MIDIA study had the needed approvals for research studies in Norway (from the Regional Ethic Committee and the Data Inspectorate) before recruitment started in the summer of 2001. Since all recruitment was based on special teaching of Norwegian public health care nurses given by the PI and a study coordinator (a pubic health care nurse working together with the PI), the recruitment started in small scale. Most of the public health care nurses in Norway started after they had got the needed information and education to voluntary recruit to MIDIA as well as being responsible for most of the blood samples taken. From 2006 the recruitment covered the whole country. In June 2007, one of the mothers of a participating baby was, however, interviewed in the biggest newspaper in Norway. She here complained about not having received good enough information about MIDIA before she and her husband had consented to participate [50, 51]. The Directorate for Health and Social Affairs then immediately decided that recruitment to MIDIA had to be stopped. Some days later it was, however, decided that new evaluation of the project had to take place according to the Norwegian Biotechnology Law, which tells that genotyping of children under the age of 18 years can only take place if there are a clear health benefit for a certain disease. During the fall of 2007, the Biotechnology Board, the Ethical Committee for the Norwegian Medical Association, the National Committee for Medical Ethics, and several experts contacted by the Directorate of Social and Health Affairs evaluated the MIDIA project. All these boards had earlier evaluated the MIDIA study (e.g., during the time of recruitment to the study). In addition, the Health Department had clearly told that children who also had developed autoantibodies in MIDIA could get health insurance. The last aspect was based on the Biotechnology Law, which Norway has had since 1994, where it is clearly told that genetic risk for a disease cannot be used by the health insurance companies. The Directorate of Social and Health Affairs found, however, genotyping in MIDIA illegal on December 10, 2007. A few days later, the Norwegian Data Inspectorate said in newspapers that all data already collected from participants in MIDIA had to be thrown away. All ended good by the Norwegian Parliament voting in June 2008. As long as the Medical Regional Committee and the Norwegian Data Inspectorate approved the MIDIA study once more, and all parents of children who already had been identified as high-risk children gave a new informed consent, research in MIDIA could continue. Close to 47,000 babies had been genotyped before December 10, 2007, and 1,047 had been identified with high-risk genotype. The parents of 706 children gave new informed consent starting from the fall of 2008 until early in 2009, Figure 1.

Norway is different from Sweden, Finland, Germany and five states in USA were no similar Biotechnology Law has given problems with genotyping of 420,000 children for The Environmental Determinants of Diabetes in the Young (TEDDY) study.

### 2.6. Ethics and Data Protection in Human Biomarker Studies

The Norwegian Biotechnology Law states the following: “genetic testing of a child under the age of 18 years is not allowed if circumstances cannot be detected that can reduce or prevent health disadvantages for the child.” Since the law came in 1994, it had only counted for clinical practice, and the MIDIA project had been run for 6.5 year before the study was stopped. Both Approval from the Regional Ethic Committee and the Norwegian Data Inspectorate were given before recruitment to the study started. The reason for a new understanding of the Biotechnology law started in the biggest newspaper in Norway. The public debate got the Notional Ethic Committe, The Etic Commitee of the Norwegian Association for Physicans, the Norwegian Biotechnology Committee and The Norwegian Directorate of Helath to evaluate the MIDIA project once more. When the Directorate of Health (directly under the Department of Health, The Norwegian Government) stopped the MIDIA project, important questions did come up.

1. Do important scientific T1D projects involving genotyping of children have to be performed elsewhere in the world? Should not Norway as one of the riches countries in the world have a certain responsibility?
2. Are not the parents able to give informed consent on behalf of their child?
3. How should health benefit be defined?
4. Is it not so that if clear health benefit has been shown, it is no longer research but part of general recommendation for public health or part of the health care system?

## 3. Results from MIDIA

### 3.1. Viral Infections

#### 3.1.1. Enterovirus

With the aim to test whether the frequency of human enterovirus RNA in faecal samples collected monthly from early infancy was associated with development of multiple islet autoantibodies in children with the highest risk HLA genotype faecal samples from 911 children that were used, 27 had developed positivity for two or more islet autoantibodies in two or more consecutive samples (case subjects) [52]. Two control subjects per case subject were matched by follow-up time, date of birth, and county of residence. The frequency of human enterovirus RNA in stool samples from case subjects before seroconversion (12.7%) did not differ from the frequency in control subjects (13.6%). There was no support for the hypothesis that faecal shedding of enteroviral RNA is a major predictor of advanced islet autoimmunity [53].

Since no association was found between children carrying the high-risk genotype and enterovirus, our aim was to assess whether genetic polymorphisms could play a role. There was no statistically significant association between other T1D associated HLA genotypes and the occurrence of human enterovirus gut infections [54]. Polymorphisms in the IFIH1 (common rs1990760 and four rare rs35667974, rs35337543, rs35744605, and rs35732034) have been convincingly associated with T1D. We therefore investigated whether the polymorphisms are associated with differences in the frequency of enterovirus RNA in blood. The genotypes of IFIH1 rs1990760 were associated with different frequencies of enterovirus RNA in blood (7.0%, 14.4%, and 9.5% bloods were enterovirus positive among children carrying the Ala/Ala, Ala/Thr, and Thr/Thr genotypes, respectively, *P* = 0.012) [55]. The common IFIH1 SNP may modify the frequency of enterovirus RNA in blood of healthy children. This effect can help explain the association of IFIH1 with T1D [56].

Since an association between T1D and enterovirus so far only had been found in Finland, we investigated enterovirus RNA in blood and islet autoimmunity. We analyzed serial blood samples collected at age of 3, 6, and 9 months and then annually from 45 children who developed confirmed positivity for at least two autoantibodies (insulin, GAD65, and/or IA-2) and 92 matched controls in the Norwegian MIDIA study. Of 807 blood samples, 72 (8.9%) were positive for enterovirus. Positivity for enterovirus RNA in blood did not predict the later induction of islet autoantibodies, but enterovirus tended to be detected more often at the islet autoantibody conversion state [57]. There was no support for the hypothesis that faecal shedding of enteroviral RNA is a major predictor of advanced islet autoimmunity.

#### 3.1.2. Parechovirus

The objective of this study was to investigate a possible association between human parechovirus infections in early infancy, diagnosed in faecal samples, and the development of islet autoimmunity in the MIDIA study [58]. A nested case-control study, including 27 children who developed islet autoimmunity (repeatedly positive for two or three autoantibodies) and 53 children matched for age and community of residence, was used. Monthly stool samples from these children were analyzed for human parechovirus. There was no significant difference in the prevalence of human parechovirus in stool samples when cases and controls were compared: 13.0 and 11.1%, respectively [59]. There was not also any difference in the number of infection episodes. In analyses restricted to samples collected 3, 6, or 12 months prior to seroconversion for islet autoantibodies, there was a suggestive association in the shortest time window of 3 months (20.8 versus 8.8%, odds ratio (OR) = 3.2, 95% CI 1.2–8.5, uncorrected *P* = 0.022). Neither was there found any Ljungan virus in the large dataset studied [60].

#### 3.1.3. Saffold Virus

We could not detect any significant associations between Saffold virus and development of islet autoimmunity (estimated OR = 2.06 (0.59–7.20)). SAFV virus genotypes 2 and 3 seem to be dominant. However, only 2.6% of samples were positive for Saffold virus, indicating that this virus is rarely present in stool (Tapia and Bøås, unpublished data).

### 3.2. Dietary Factors and T1D Risk

Breastfeeding protected against enterovirus [61], and breastfeeding for a period for more than 12 months delayed disease progression from autoimmunity to clinical T1D [62]. No differences were found in the MIDIA study for the time point of introducing solid food—but it seemed important that the mother still was breast feeding.

In the MIDIA study, a cohort design was used for assessing whether body mass index (BMI) before pregnancy and weight gain during pregnancy predicted the risk of islet autoimmunity in 885 children. 36 of the children developed autoimmunity, of who 10 developed T1D. Both maternal body mass index (BMI) before pregnancy and weight gain > or = 15 kg predicted increased risk for islet autoimmunity with significant hazard ratio (HR) at 2.5 for both situations [63].

### 3.3. Wheezing in Early Infancy

When a cohort of 42 cases and 843 noncases in MIDIA was studied, self-reported “pneumonia, bronchitis, or RS-virus” had HR at 3.5, *P* = 0.001, for development of autoimmunity before 4 years of age [64]. Also a Swedish study with data collected at the age of 2.5 years found that wheezing during the first year of life was significantly associated with islet autoimmunity [65].

### 3.4. *Enterobius vermicularis*

*Enterobius vermicularis* still seems to be common during childhood. However, pinworm infections seem to be uncommon in children younger than 2 years and have the highest prevalence in children older than 5 years of age (34%). Increased number of siblings was linked to more infections, and there were fewer infections in the children with the high-risk genotype [66]. A possible association between current pinworm infections and food allergy was found (OR = 2.9 (1.1–8.0)) and needs to be studied in a larger material [67].

### 3.5. The Diversity of the Data Obtained in the Different National Cohort Studies

As appearing from what is found above, different factors have been reported to confer T1D susceptibility in the different national prospective cohorts, leaving a number of holes and a troubling lack of consistency in the findings to date. It is likely that the results have been confounded by imprecise assessment of dietary exposure, recall bias, failure to assess dietary exposures at very early ages, different definitions of exposure, and small sample sizes. To solve these issues was the background for the large collaborative study TEDDY [68]. 420,000 newborns were screened for human leukocyte- (HLA-) conferred genetic risk for T1D; 21,589 were HLA eligible, and 8,668 joined the TEDDY study (40% participation rate). As of September 2014, 2613 families have withdrawn (28% participation rate of the eligible) [69, Personal communication, the TEDDY group]. Although so much efforts and funding have been given to the TEDDY study, probably also here that many reports will show too little power to conclude for a specific environmental factor.

### 3.6. A New Collaborative Effort

Most probably there are already enough prospective cohort data collected or under collection to identify the environmental trigger(s) of T1D. New valuable information about factors and their contribution associated with the development of *β*-cell autoimmunity and progression to T1D could be achieved by a hurge international collaborative effort. It would then be possible to integrate demographic, genetic, autoimmune, and exposure data from the existing cohorts in Finland, Norway, Sweden, Germany, and Denver, Colorado.

## 4. The DIPP Study

The Type 1 Diabetes Prediction and Prevention (DIPP) study in Finland is a population-based long-term clinical follow-up study established in 1994, 1995, and 1997 in three university hospitals in Finland (Turku, Oulu, and Tampere, respectively) to understand the pathogenesis of T1D, predict the disease, and find preventive treatment [70, 71]. Both recruitment and follow-up of children in this study have since then been constantly ongoing; 186,000 cord blood samples have been genotyped so far. Families with a newborn baby carrying a DR-DQ genotype associated with increased risk for T1D (approximately 10% of all infants) are invited to participate in regular follow-up at the age of 3, 6, 9, 12, 18, and 24 months and thereafter once a year until the age of 15 years or until T1D is diagnosed, Table 2. Clinical details including maternal diet during pregnancy and lactation and child's diet starting from the age of 3 months are recorded, blood samples are collected, and serum autoantibodies associated with development of T1D are measured. In the DIPP study about 750 children have developed multiple islet autoantibodies, and more than 300 of these have progressed to clinical T1D.

## 5. The DiPiS Study

The Diabetes Prediction in Skåne (DiPiS) study is a population-based long-term follow-up study in Skåne, the southernmost region in Sweden representing 1.2 million inhabitants, 12,000 newborns per year, and nearly 100 children below 18 years of age diagnosed with T1D every year. In 2000–2004, more than 35,000 (70% of all newborns) were screened at birth for T1D high risk HLA, and 25,000 filled out a questionnaire on gestational and perinatal health [72], Table 3. Nearly 6,000 children at increased risk for T1D were offered follow-up and 4,200 are followed since two years of age, 82 have developed two or more islet autoantibodies, and 33 (40%) have gone on to a clinical diagnosis of T1D. The DiPiS children will be followed until 15 years of age.

## 6. The BABYDIAB Study

The BABYDIAB is a study from birth in 1,650 children born to a mother or father with T1D. Recruitment began in 1989 and ended in 2000. All children (840 boys, 810 girls) were recruited in Germany [73–76], Table 4. The population is not population based, and 97% of the families are of German or of European descent. Islet autoantibodies directed against insulin (IAA), glutamic acid decarboxylase (GAD), insulinoma-associated protein 2 (IA-2), and Zinc transporter 8 (ZnT8) are tested at all scheduled visits and every 6 months in children positive for islet autoantibodies. The median follow-up from birth to last sample in BABYDIAB is 11.7 years [45]. HLA genotyping has not been any inclusion criteria but has been performed later on for scientific purposes.

## 7. The DAISY Study

Between December 1993 and October 2004. The Diabetes Autoimmunity Study In the Young (DAISY) screened for T1D susceptibility HLA-DR, DQ genotypes and tested over 33,000 newborns from the general population of Denver, Colorado. The study population was representative of the general population of the Denver Metropolitan Area and included children classified by their mothers as non-Hispanic white (58%), Hispanic (28%), African American (7%), Asian American (2%), or biracial/others (5%). Newborns were categorized into four risk groups: (1) high T1D risk, 20-times higher than in the general population (HLA-DRB1^*^ 03/04,DQB1^*^ 0201/0302 genotype and negative for DRB1^*^ 0403); (2) moderate T1D risk, 3–7-times higher than in the general population (HLA-DR,DQ 4/4, 1/4, 8/4, and 9/4 (the DR4 haplotype carrying DQB1^*^ 0302), and DR3/3); (3) average T1D risk, similar to that for the general population (HLA-DRB1^*^ 03/x or ^*^ 04/x), and (4) low diabetes risk—all others. The combination of high- (2.1%) and moderate-risk genotypes (7.5%) was present in 9.6% of the general population. All high-risk children and selection of those at moderate or low risk were invited to participate in the follow-up [77–85], Table 5. Families with a member with T1D were identified using The Barbara Davis Centre for Childhood Diabetes in Denver, other diabetes care clinics, the Colorado T1D Registry, and newspaper publicity.

### 7.1. How to Find the Environmental Triggers of T1D without New Prospective Cohorts?

The synthesis of multiple data sources to increase our understanding of a research field is an essential part of the scientific method. In the case of T1D, a close collaboration between the PI and close coworkers of existing cohorts (MIDIA, DIPP, DiPiS, BABYDIAB, and DAISY) would be a very vulnerable recourse in identification of the environmental trigger(s) of T1D. Given the diversity of systems and formats in which the data are currently stored, it is very difficult to create association between the results from the different studies. Several manual steps would be necessary to collect the data from various sources, transform them into a common format, and prepare them for a single kind of analysis. Such an ad hoc process would be not only complex and time consuming, but also hard to reuse and benefit from further studies of different analysis. To solve this interoperability issues, it would be much better to create a combined database that acts as a central repository, providing methods to store and retrieve data quickly and efficiently. Having a unified view on all the available information will allow the application of advanced analysis methods. It will also make it much easier to add further results, as it will only be necessary to adapt them to the common data format. The database would hold information, relative to all kinds of risk factors, for more than 20 000 children at various grades of increased HLA-conferred risk for T1D, followed from their early infancy (3 months) until seroconversion for *β*-cell specific autoantibodies and, in many cases, to clinical onset of the disease. To create a harmonized representation of the data, the start would be collecting and studying the results provided by each of the partners in this huge cohort project. Detailed documentation will have to be created on the factors presenting each of the cohort studies: how they are stored and how they relate to each other. The next step would be to design a database solution that aggregates those factors and that satisfies the requirements of the data analysis tasks to be performed. To incorporate and update the results on a regular basis an automated information integration process for each of the cohort data sources should be incorporated into the database.

In conclusion, based on international experiences with cohort studies, and with the relatively small participation rate in the TEDDY study, it is probably now a better idea with a new international effort to find the environmental trigger(s) of T1D. All the ongoing cohort studies will give a unique resource for collaboration. Performing large-scale integrative analysis on the combined database of available and incoming cohort data will give new insights and unfold complex relationships between the factors that determine the pathogenesis of the T1D.

## Figures and Tables

**Figure 1:**
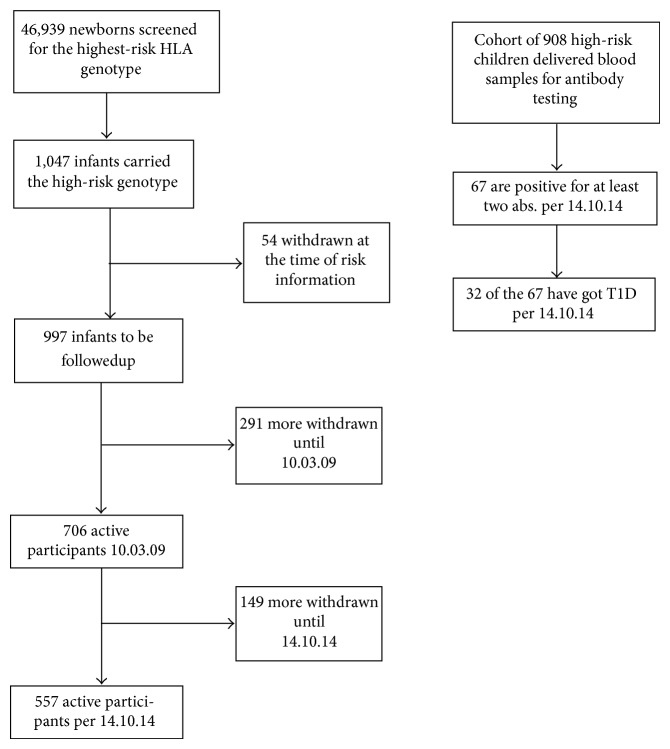
The first box to the left shows total number of children genotyped for the high-risk genotype in MIDIA. Of the 997 children who had parents participating in MIDIA, 908 delivered blood samples more than once for autoantibody testing. The rest of participants delivered stool samples and questionnaires. 706 children had parents who had given a new informed consent in 2008/2009. Abs.: autoantibodies.

**Table 1:** HLA typing and time points for follow-up with blood samples in MIDIA.

(a) Eligible HLA type	DRB1^*^03-DQA1^*^05-DQB1^*^02/DRB1^*^0401-DQA1^*^03-DQB1^*^0302

(b) Time points for blood samples	3, 6, 9, and 12 months, thereafter annually If positivity for one autoantibody, a new blood sample after 6 months. If positivity for two or three autoantibodies, a new samples after 3 months

(c) Questionnaires	Collected at 3, 6, 9, and 12 months and thereafter annually

(d) Stool samples	Collected each month from 3 months to 3 years of age

**Table 2:** HLA typing and time points for follow-up in DIPP.

(a) Eligible HLA types	DQB1^*^02/DQB1^*^0302
	DQB1^*^0302/X (X # DQB1^*^02, 0301, 0602/0603)

(b) Time points for blood samples	Autoantibodies are measured in Tampere and Oulu at 3, 6, 18, and 24 months and then annually. In Turku every 3 months until 2 years, and then every 6 months until the age of 14 years

(c) Questionnaires	Filled out at the clinical visits at 3, 6, 18, and 24 months, and then annually

(d) Stool samples	Parents are asked to send it from 3 months to 3 years

**Table 3:** HLA typing and time points for follow-up in DiPiS.

(a) Eligible HLA types	DQB1^*^02/DQB1^*^0302
	DQB1^*^0302/X (X # DQB1^*^02, 0301, 0602/0603)

(b) Time points for blood samples	Autoantibodies are measured in every second year

(c) Questionnaires	Filled just after the children were born, gestational and perinatal health

(d) Stool samples	Not asked for

**Table 4:** HLA typing and time points for follow-up in BABYDIAB.

(a) No HLA typing required for recruitment and to be found eligible for follow-up	

(b) Time points for blood samples: 9 months, 2 years, 5 years, 8 years, 11 years, 14 years, 17 years, and 20 years	

(c) Questionnaires were asked for at 9 months and at 2 years with respect to breastfeeding	

(d) Stool samples, not asked for	

**Table 5:** HLA typing and time points for follow-up in DAISY (general population).

(a) Eligible HLA types^†^	DRB1^*^03-DQB1^*^02/DRB1^*^04-DQB1^*^0302, and negative for DRB1^*^0403
	DRB1^*^04-DQB1^*^0302/DRB1^*^04-DQB1^*^0302, and negative for DRB1^*^04034
	DRB1^*^03-DQB1^*^02/DRB1^*^03-DQB1^*^02
	DRB1^*^04-DQB1^*^0302/DRB1^*^01-DQB1^*^0501, and negative for DRB1^*^0403
	DRB1^*^04-DQB1^*^0302/DRB1^*^08-DQB1^*^0402
	DRB1^*^04-DQB1^*^0302/DRB1^*^09-DQB1^*^0303

(b) Time points for blood samples	9, 15, and 24 and thereafter annually

(c) Questionnaires	Filled out at the clinical visits at 9, 15, and 24 months of age, and thereafter annually

(d) Rectal swabs and salvia:	At 9, 15, and 24 months of age, and annually thereafter

^†^No HLA typing for siblings of a child with T1D.
